# Carotid plaque ulceration: unquantified predictor of stroke

**DOI:** 10.1093/bjsopen/zrad058

**Published:** 2023-06-23

**Authors:** Luke C Smith, Jonathan P Funnell, Toby Richards, Lawrence M J Best

**Affiliations:** Intensive Care Unit, Royal Hampshire County Hospital, Winchester, UK; Neurosurgery Department, St George's Hospital, London, UK; Division of Surgery, The University of Western Australia (M581), Perth, Western Australia, Australia; UCL Division of Surgery and Interventional Science, Royal Free Hospital, London, UK

## Introduction

The decision to operate on patients with carotid artery disease is made principally using the degree of carotid artery stenosis. Evidence linking the severity of carotid artery stenosis and the future risk of stroke was derived from the seminal trials reported in the 1990s—the European Carotid Surgery Trial,^1^ the North American Symptomatic Carotid Endarterectomy Trial,^2^ the Veteran Affairs Trial,^3^ and the pooled results of these trials presented in Rothwell *et al*.^4^. Decisions on whether to operate on patients are still based primarily on these now historical data together with consideration of factors such as age, sex, time since last cerebrovascular event, types of symptoms, and co-morbidities^5^. Currently, no other features of plaque morphology besides carotid stenosis are formally used to guide decision-making about whether to operate.

One risk factor that may be useful in a more accurate treatment algorithm is carotid plaque ulceration. *Post*-*hoc* analysis of the European Carotid Surgery Trial highlighted that carotid plaque ‘irregularity’ was associated with increased risk of stroke^6^. This was based on the now obsolete practice of carotid angiography. Carotid plaque ulceration can be detected on routinely performed ultrasound, magnetic resonance angiography (MRA), or CT angiography, but its significance for future stroke risk is unclear. A systematic review and meta-analysis was performed to assess the current evidence for carotid plaque ulceration as a predictor of stroke.

## Methods

A systematic review and meta-analysis of prospective or retrospective studies registered on PROSPERO (registration number: CRD42020111190) was performed on 25 March 2020. Embase, MEDLINE, and Web of Science electronic databases were searched, returning 10 166 non-duplicate references. The search strategy used is presented in *Fig. S1*. Full-text review was undertaken for 274 studies of which 16 studies provided data on 4246 patients, which were included in a meta-analysis (*Fig. S2*). Carotid ulceration was identified either by carotid ultrasound (2876 patients) or catheter angiography (1370 patients). Only studies using non-catheter angiography methods (all of which only reported data for ultrasound) are reported here, as catheter angiography is no longer relevant for clinical practice. The endpoints assessed were stroke, and stroke or transient ischaemic attack (TIA), either as a dichotomous or time-to-event outcome, depending on how study authors reported outcomes. Review Manager 2014 (Revman 5.3) was used to manage the data and perform the analysis.

## Results

Overall, 21.6 per cent of patients had carotid ulceration, ranging from 4.5 to 100 per cent by study. The definition of carotid ulceration varied between studies. Seven studies provided no details, four studies defined ulceration using the Moore *et al*.^7^ criterion (radiologist could clearly identify a discrete ulcer), and the remaining five studies had individual definitions for ulceration: Madani *et al*.^8^ defined ulceration quantitatively as a break of 1 mm or more in the plaque surface, Nicolaides *et al*.^9^ as a defect of 2 × 2 mm in the surface of the plaque, and the remaining three studies gave qualitative definitions (*Table S1* and *Table S2*).

**Fig. 1:**
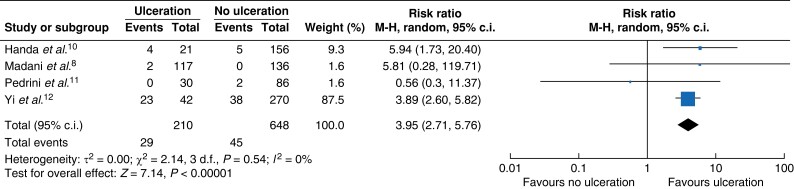
Forest plot of results for stroke at maximal follow-up comparing patients with carotid artery stenosis with ulceration *versus* those without ulceration at baseline M-H, Mantel-Haenszel method.

**Fig. 2:**
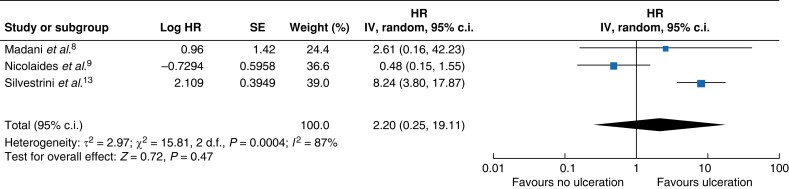
Forest plot of results for time to stroke comparing patients with carotid artery stenosis with ulceration *versus* those without ulceration at baseline SE, standard error; IV, inverse variance.

Four studies including 648 patients reported stroke at maximal follow-up. In these studies, which included a mix of symptomatic and asymptomatic patients, carotid plaque ulceration was associated with an approximately four times greater likelihood of stroke (risk ratio 3.95 (95 per cent c.i. 2.71 to 5.76)) (*Fig. 1*). Three studies including 1995 patients reported stroke as a time-to-event outcome, which showed a non-significant association (HR 2.20 (95 per cent c.i. 0.25 to 19.11)) (*Fig. 2*).

Two studies reported stroke or TIA at maximal follow-up and two studies reported time to stroke or TIA. Neither of these showed evidence of an association between carotid plaque ulceration and stroke or TIA.

## Discussion

In patients with carotid artery stenosis, carotid plaque ulceration can be identified in approximately one in five patients by ultrasound. Overall, the presence of carotid plaque ulceration was associated with an increased risk of future stroke. However, the significance of these data for individual patients is unclear and this study has a number of limitations, namely the small patient cohort, the timing of imaging as it is likely that percentage ulceration would be higher at the time of a cerebrovascular event, and also the use of ultrasound as the imaging modality of choice given the conflicting evidence regarding its diagnostic accuracy for ulceration. Some studies suggest that ultrasound has high sensitivity and specificity^14,15^ for diagnosing plaque ulceration, whereas others conclude that ultrasound has a low sensitivity (23–47 per cent)^16–18^.

Further work is required to assess the association of carotid plaque ulceration and patient outcomes. Plaque ulceration may be more reliably detected using contrast-enhanced ultrasound, multidetector computed tomography, and MRA sequencing using a consistent definition of ulceration that has been standardized across these imaging modalities. A large triple imaging modality comparison study of symptomatic patients with sub-threshold stenosis and asymptomatic patients with follow-up for stroke/mortality in patients treated with medical therapy alone may be required. If there is a clear association between ulceration and risk of stroke, future guidelines may incorporate plaque ulceration in a more comprehensive risk scoring system for predicting future stroke in patients with carotid artery disease.

In patients with carotid stenosis about one in five have evidence of plaque ulceration, which is broadly associated with increased risk of stroke, but the importance for the individual patient is still unclear. The presence of plaque ulceration should be documented in all patients with carotid stenosis and further research completed to determine its importance as a risk factor for patient morbidity and mortality.

## Supplementary Material

zrad058_Supplementary_Data

## Data Availability

Data are available on request from the authors.
