# Supplementary material for: Carotid plaque ulceration: unquantified predictor of stroke
Source: BJS Open. 2023 Jun 23;7(3):zrad058. doi: 10.1093/bjsopen/zrad058 (PMC10289828; doi:10.1093/bjsopen/zrad058)
Supplement: zrad058_Supplementary_Data [file zrad058_supplementary_data.docx]

Carotid plaque ulceration: an unquantified predictor of stroke

Supplementary Materials

Luke C. Smith ^a*^ , Jonathan P. Funnell ^b^ , Toby Richards ^c^ , Lawrence M. J. Best ^d^

^a^ Royal Hampshire County Hospital, Intensive Care Unit, Romsey Road Winchester, SO22 5DG [lukesmith777@doctors.org.uk](mailto:lukesmith777@doctors.org.uk)

^b^ Addenbrooke’s Hospital, Hills Rd, Cambridge CB2 0QQ, UK [jonathan.funnell@nhs.net](mailto:jonathan.funnell@nhs.net)

^c^ Division of Surgery, The University of Western Australia (M581), 35 Stirling Highway, 6009 Perth, Australia [toby.richards@uwa.edu.au](mailto:toby.richards@uwa.edu.au)

^d^ UCL Division of Surgery and Interventional Science, Royal Free Hospital, 9^th^ Floor (East), 10 Pond Street, London, NW3 2PS, UK [Lawrence.best.11@ucl.ac.uk](mailto:Lawrence.best.11@ucl.ac.uk)

*Corresponding Author:

Luke Smith

Email: [lukesmith777@doctors.org.uk](mailto:lukesmith777@doctors.org.uk), ORCID ID: 0000-0001-6648-5854

**Supplementary Materials - Index**

| **Supplementary Figures** |  |
| --- | --- |
| Figure S1: Search strategies used A) Embase Medline B) Web of Science | *Page 2* |
| Figure S2: PRISMA flow diagram showing the paper selection method | *Page 3* |
| **Supplementary Tables** |  |
| Table S1: Summary of findings of studies with time to event data | *Page 4* |
| Table S2: Summary of findings of studies with dichotomous outcomes | *Page 5* |
|  |  |

**Supplementary Figures**

1. **Ovid search strategy for embase and medline:**

| Search Number | Search Strategy |
| --- | --- |
| #1 | Cerebral infarction.mp. OR exp cerebral infarction/ |
| #2 | Brain infarction.mp. OR exp Brain infarction/ |
| #3 | Exp brain ischemia/ OR exp ischemic attack, transient/ OR transient ischaemic attack.mp. |
| #4 | Transient ischemic attack.mp. |
| #5 | TIA.mp. |
| #6 | Exp stroke/ OR exp stroke, lacunar/ |
| #7 | stroke*.mp. |
| #8 | exp carotid artery injuries/ OR exp carotid intima-media thickness/ OR exp endarterectomy, carotid/ OR exp carotid artery diseases/ OR exp carotid stenosis/ OR exp carotid artery, internal/ OR exp carotid artery, common/ OR exp carotid arteries/ OR carotid*.mp. |
| #9 | Exp ulcer/ OR ulcer*.mp. |
| #10 | Exp plaque, atherosclerotic/ OR plaque*.mp. |
| #11 | 1 OR 2 OR 3 OR 4 OR 5 OR 6 or 7 |
| #12 | 9 OR 10 |
| #13 | 8 AND 11 AND 12 |

1. **Search strategy for Web of Science:**

| Search Number | Search Strategy |
| --- | --- |
| #1 | TS= (cerebral infarction OR brain infarction OR TIA OR transient ischaemic attack OR stroke) |
| #2 | TI= (cerebral infarction* OR brain infarction* OR TIA OR transient ischaemic attack OR transient ischemic attack OR stroke) |
| #3 | TS= (carotid artery OR carotid stenosis) |
| #4 | TI= (carotid arter* OR carotid stenosis) |
| #5 | TS= (ulcer OR plaque) |
| #6 | TI= (ulcer* OR plaque*) |
| #7 | #2 OR #1 |
| #8 | #4 OR #3 |
| #9 | #6 OR #5 |
| #10 | #9 AND #8 AND #7 |

**Figure S1:** Search strategies used A) Embase and Medline B) Web of Science


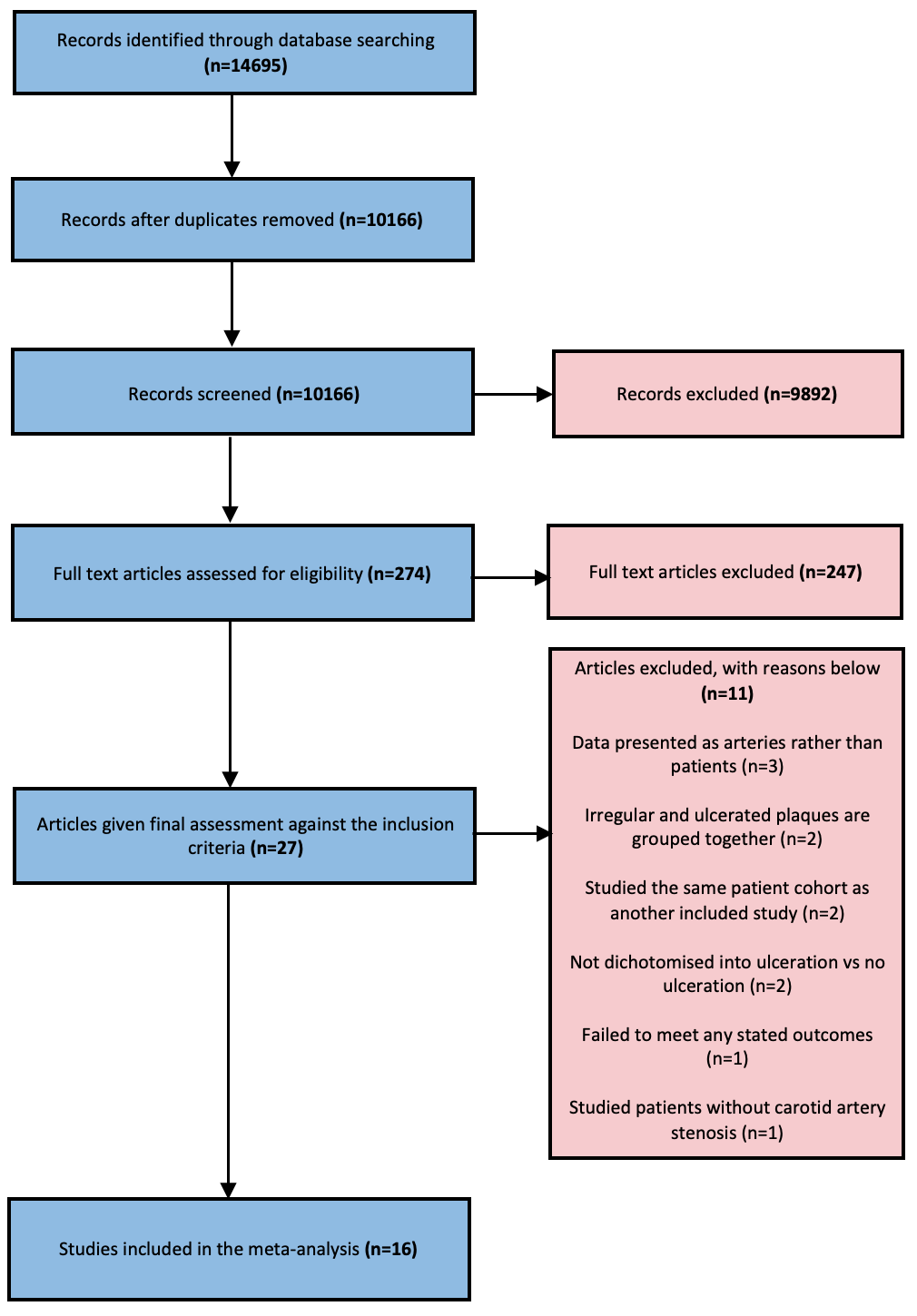


**Figure S2:** PRISMA flow diagram showing the paper selection method

**Supplementary Tables**

**Table S1:** Summary of findings of studies with time to event data

| Author | Imaging used for carotid plaque assessment | Ulceration definition | Total number of patients | No. of patients meeting inclusion criteria | No. with carotid plaque ulceration at baseline | No. without carotid plaque ulceration at baseline | No. Symptomatic at baseline | No. asymptomatic at baseline |
| --- | --- | --- | --- | --- | --- | --- | --- | --- |
| Eliasziw et al. | Catheter angiogram | ‘Radiographic criteria of ulcer niche, seen in profile as a crater  from the lumen into a stenotic plaque and (when visible) double density on en face view’ | 659 | 331 assigned to medical treatment | 113 | 218 | 331 | 0 |
| Handa et al. | Duplex Ultrasound | Ulceration defined as per Moore et al. | 214 | 177 patients with carotid atherosclerosis | 21 | 156 | NR | NR |
| Madani et al. | 3D Ultrasound | ‘a continuous contour showing focal depression, a well-defined break in the surface of 1 mm or more across, a well-defined back wall at the base of the depression, and an anechoic area within the plaque which extended to the surface and was 1 mm or more deep’ | 253 | 253 | 117 | 136 | 46 | 207 |
| Nicolaides et al. | Duplex Ultrasound | ‘A defect 2 x 2 mm on the surface of the plaque shown by color flow or power Doppler to be communicating with the vessel lumen.’ | 1121 | 1121 | 101 | 1020 | 0 | 1121 |
| Silvestrini et al. | Duplex Ultrasound | NR | 621 | 621 | 28 | 593 | 0 | 621 |

Duplex Ultrasound= Doppler and B mode ultrasound

Catheter angiogram=X ray angiography

NR= Not reported

**Table S2:** Summary of findings of studies with dichotomous outcomes

| Author | Imaging used for carotid plaque assessment | Ulceration definition | Total no. of patients | No. of patients meeting the inclusion criteria of this study | No. with carotid plaque ulceration at baseline | No. without carotid plaque ulceration at baseline | No. symptomatic at baseline | No. asymptomatic at baseline |
| --- | --- | --- | --- | --- | --- | --- | --- | --- |
| Bogousslavsky et al 1981.^15^ | Catheter angiogram | NR | 23 | 23 | 7 | 16 | NR | NR |
| Bogousslavsky et al 1985.^16^ | Catheter angiogram | NR | 7 | 7 | 2 | 5 | 7 | 0 |
| Dixon et al.^17^ | Catheter angiogram | Ulceration defined as per Moore et al.^31^ | 141 | 141 | 141 | 0 | 0 | 141 |
| Durward et al.^18^ | Catheter angiogram | NR | 73 | 73 | 23 | 50 | 0 | 73 |
| Giannoni et al.^20^ | Duplex Ultrasound | ﻿‘Plaques  with a surface excavation or a sharp interruption of the  echoes’ | 75 | 75 | 16 | 59 | 0 | 75 |
| Harward et al.^22^ | Catheter angiogram | Ulceration defined as per Moore et al.^31^ | 79 | 79 | 79 | 0 | NR | NR |
| Matzsch et al.^24^ | Catheter angiogram | ﻿‘Outlines of the plaque was not smooth and uninterrupted’ | 609 | 578 could be followed up | 71 | 507 | NR | NR |
| Pedrini et al.^26^ | Duplex Ultrasound | NR | 491 of which 289 were followed up | 116 (after removal of ‘not specified’ plaque morphology subset) | 30 | 86 | 62 | 54 |
| Schroeder et al.^27^ | Catheter angiogram | Ulceration defined as per Moore et al.^31^ | 185 | 125 (10 patients removed who were categorized as uncertain plaque morphology) | 87 | 38 | 0 | 135 |
| Singh et al.^29^ | Duplex Ultrasound, MRA,  CTA or conventional angiogram | NR | 214 | 214 | 23 | 191 | 0 | 214 |
| Yi et al.^30^ | Duplex Ultrasound | NR | 312 | 312 | 42 | 270 | 312 | 0 |

MRA= Magnetic resonance angiography, CTA= Computed tomographic angiography

NR= Not reported
